# BAG5 Protects against Mitochondrial Oxidative Damage through Regulating PINK1 Degradation

**DOI:** 10.1371/journal.pone.0086276

**Published:** 2014-01-24

**Authors:** Xuejing Wang, Jifeng Guo, Erkang Fei, Yingfeng Mu, Shuang He, Xiangqian Che, Jieqiong Tan, Kun Xia, Zhuohua Zhang, Guanghui Wang, Beisha Tang

**Affiliations:** 1 Department of Neurology, Xiangya Hospital, Central South University, Changsha, Hunan, People's Republic of China; 2 State Key Laboratory of Medical Genetics, Changsha, Hunan, People's Republic of China; 3 Laboratory of Molecular Neuropathology, National Laboratory for Physical Sciences at Microscale and School of Life Sciences, University of Science and Technology of China, Hefei, Anhui, People's Republic of China; 4 Human Key Laboratory of Neurodegenerative Disorders, Central South University, Changsha, Hunan, People's Republic of China; 5 Neurodegenerative Disorders Research Center, Central South University, Changsha, Hunan, People's Republic of China; Emory University, United States of America

## Abstract

Mutations in PTEN-induced kinase 1 (PINK1) gene cause *PARK6* familial Parkinsonism, and loss of the stability of PINK1 may also contribute to sporadic Parkinson's disease (PD). Degradation of PINK1 occurs predominantly through the ubiquitin proteasome system (UPS), however, to date, few of the proteins have been found to regulate the degradation of PINK1. Using the yeast two-hybrid system and pull-down methods, we identified bcl-2-associated athanogene 5 (BAG5), a BAG family member, directly interacted with PINK1. We showed that BAG5 stabilized PINK1 by decreasing the ubiquitination of PINK1. Interestingly, BAG5 rescued MPP^+^- and rotenone-induced mitochondria dysfunction by up-regulating PINK1 in vitro. In PINK1-null mice and MPTP-treated mice, BAG5 significantly increased in the substantia nigra pars compacta (SNpc) although PINK1 was decreased. Our findings indicated that BAG5, as a key protein to stabilize PINK1, is a promising therapeutic tool for preventing mitochondrial dysfunction following oxidative stress.

## Introduction

It is well recognized that Parkinson's disease (PD) is a primarily sporadic occurring neurodegenerative disorder of advanced age. However, with increasing awareness of the importance of genetic factors involved in the disease, interest has focused on the role of the several genes identified that lead to a hereditary parkinsonian disorder for over ten years [1], [2]. Several mutations in PTEN-induced putative kinase 1 (PINK1) gene have been reported to be associated with recessive PD, which was considered as the second common virulence gene besides Parkin [3]–[6]. The encoded protein PINK1 is a 581 amino acid protein with a mitochondrial localization signal (MLS) and a functional serine/threonine kinase domain, which was identified to be degraded by the UPS [4], [7], [8]. Previous studies have demonstrated that degradation of PINK1 by ubiquitin proteasome system (UPS) is regulated by Parkin through a direct interaction between them [9]–[14]; and expression of wild-type DJ-1 increased steady-state levels of PINK1, whereas expression of DJ-1A39S reduced steady-state levels of PINK1 [15]. PINK1 protects against oxidative stress-induced apoptosis by directly phosphorylating its downstream effector, TNF receptor-associated protein 1 (TRAP1) [16]; and PINK1 modulates the levels of phosphorylated HtrA2, thereby contributing to an increased resistance of cells to mitochondrial stress [10], [17]. These results indicated that the interactions between PINK1 and its upstream or downstream proteins may play an important roles in the pathogenesis of PD.

The BAG (Bcl-2 associated athanogene) family is a group of multifunctional proteins that can function as the cochaperones of Hsp70s [18]. Members of the BAG protein family all contain BAG domain (BD), which mediates direct interaction with the ATPase domain of Hsp70/Hsc70 molecular chaperones [18], [19]. BAG5 that contains five BDs is a unique member of the BAG family. Little is known about the functions of BAG5 other than its important role in PD. Previous study showed that BAG5 inhibited both Parkin E3 liase and Hsp70 chaperone activities thereby enhancing dopaminergic neuron degeneration [20]. However, a recent study demonstrates that BAG5 can function as the nucleotide exchange factor of Hsp70 for the enhancement of protein refolding [21].

In this study, we demonstrated BAG5 directly interacted with PINK1, and regulated PINK1 degradation via UPS. In addition, BAG5 protected mitochondria against MPP^+^- and rotenone-induced oxidative. Further investigations revealed decrease of PINK1 levels under MPP^+^ treatment or suppression of PINK1 expression resulted in up-regulation of BAG5 in vitro or in vivo. These data suggest that the interaction between BAG5 and PINK1 may play an important role in the pathogenesis of PD.

## Results

### PINK1 interacts with BAG5 in vitro and in vivo

**Figure 1:**
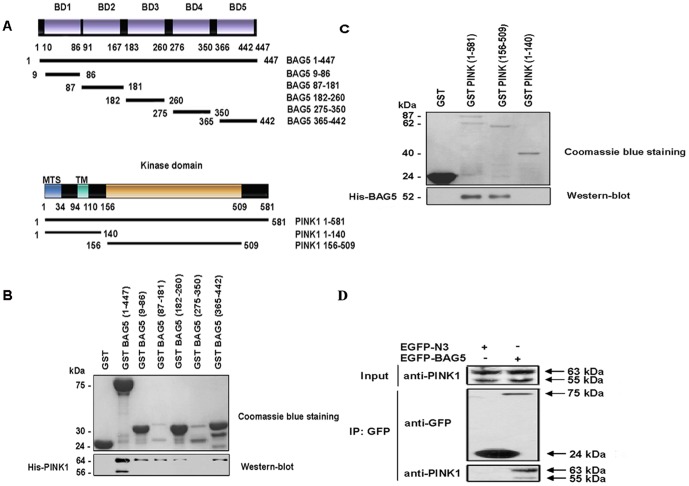
PINK1 interacted with BAG5 in vitro and in vivo. **A:** Various deletion plasmid constructs of BAG5 and PINK1. BAG5 was composed of five BDs (BAG domain), designated as BD1, BD2, BD3, BD4 and BD5. PINK1 was composed of the MTS (mitochondrial targeting sequence), MT (transmembrane domain) and Kinase domain. **B:** PINK1 interacted with the BAG domains of BAG5 in vitro. Coomassie blue staining showed the amount of BAG5 fusion protein used in each pull down, and the western blot demonstrated GST-BAG5(1–447), GST-BAG5(9–86), GST-BAG5(87–181), GST-BAG5(182–260) and GST-BAG5(365–442) all interacted directly with His-PINK1, whereas GST-alone and GST-BAG5(275–350) did not. **C:** BAG5 interacted with the kinase domain of PINK1 in vitro. Western blot analysis demonstrated GST-PINK1(1–581) and GST-PINK1(156–509) all interacted directly with His-PINK1, whereas GST-alone and GST-PINK1(1–140) did not. **D:** Co-IP of endogenous PINK1 and overexpressed EGFP-tagged BAG5 in HEK-293 cells.

To identify potential PINK1 partners, we employed the yeast two-hybrid screen. The full-length of human PINK1 cDNA (1–1746 bps) was cloned into pGBKT7 vector (Matchmaker III from Clontech) and verified by sequencing and immunoblot analysis. After sequential transformation of the pGBKT7-PINK1 bait and the fetal brain pACT2 cDNA library, two clones were isolated. Sequencing of the prey cDNA and subsequent bioinformatic analysis showed that one of these proteins was BAG5, a member of the BAG family. To confirm the yeast two-hybrid data, we performed GST pull down assays to detect the interaction between PINK1 and BAG5 in vitro. To identify the region of BAG5 that mediates the interaction with PINK1, we generated various deletion constructs of BAG5, which were designed to delineate the binding activity of each BD of BAG5 (Fig. 1A). GST-BAG5 (1–447), GST-BAG5(9–86), GST-BAG5(87–181), GST-BAG5(182–260) and GST-BAG5(365–442) all pulled down the PINK1-FL, but GST-alone and GST-BAG5(275–350) did not (Fig. 1B). In the converse experiment, we created the mitochondrial targeting domain and kinase domain of PINK1 to determine the domains of PINK1 required for the interaction(Fig. 1A). The kinase domain of PINK1 was necessary and sufficient for the interaction, whereas the mitochondrial targeting domain of PINK1 was expendable(Fig. 1C). We next performed co-immunoprecipitation experiments to further examine the interaction between PINK1 and BAG5 in mammalian cells. HEK-293 cells were co-transfected with HA and EGFP-tagged BAG5. After immunoprecipitation with a rabbit polyclonal anti-GFP antibody, the immunoprecipitants were subjected to immunoblot analysis with a mouse monoclonal anti-GFP or anti-HA antibody. The results showed that EGFP-tagged BAG5 specifically co-immunoprecipitated endogenous PINK1 (Fig. 1D).

### BAG5 specifically inhibits degradation of PINK1

**Figure 2:**
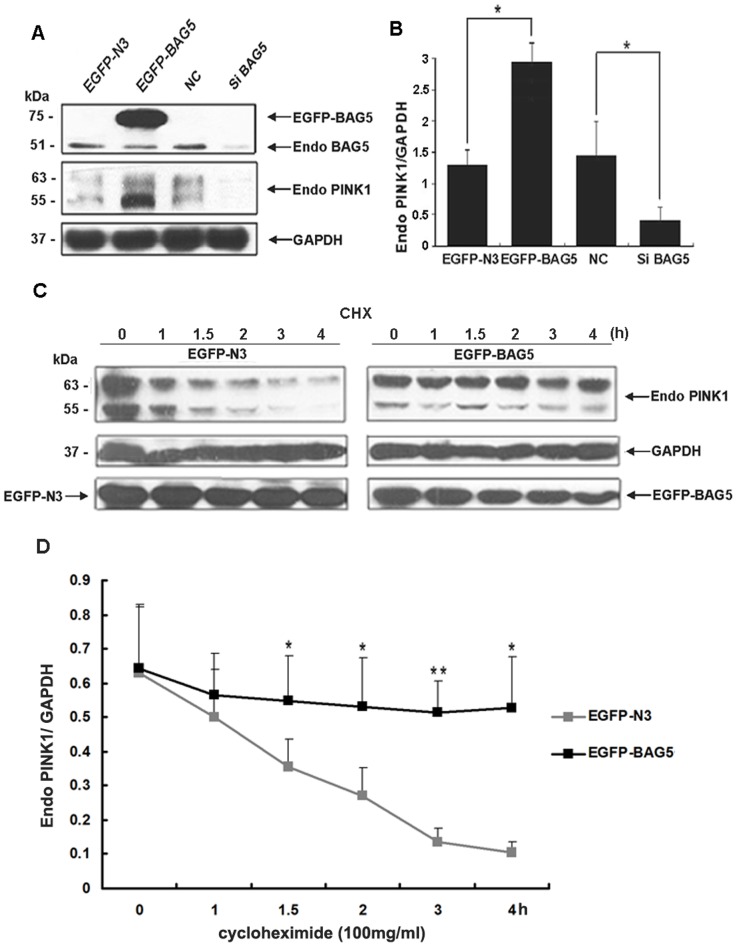
BAG5 inhibited PINK1 degradation. **A:** HEK-293 cells were transfected with EGFP-N3, EGFP-BAG5, negative control siRNA (NC) or BAG5 siRNA for 24 h or 48 h. Cells extracts were analyzed by immunoblotting using the specified antibodies. PINK1 levels were increased in EGFP-BAG5-expressing cells, whereas knockdown of BAG5 decreased the PINK1 levels. **B:** Quantitative data from A were shown. The data represented mean ± SE (n = 3; *, P<0.05; Student's t test), GAPDH served as a loading control. **C:** HEK-293 cells were transfected for 24 h with either EGFP-N3 or EGFP-BAG5, after which they were incubated for the indicated times in the presence of 100 mg/ml CHX. Before the addition of CHX or after the indicated times, cells were resuspended in lysis buffer, and the proteins were analyzed by immunoblotting using anti-PINK1 antibody. **D:** Similar results were obtained in two independent experiments, quantitative data from three independent experiments were shown. The results were indicated mean ± SE (n = 3; *, P<0.05; **, P<0.01; Student's t test).

To determine the direct function of the association between BAG5 and PINK1, HEK-293 cells were transfected with plasmids expressing either EGFP-BAG5 or EGFP alone. Exogenous PINK1 levels were increased in cells expressing EGFP-BAG5 compared with those expressing EGFP alone (Fig. 2A & 2B). Then we performed siRNA-mediated suppression of BAG5, and found that knockdown of BAG5 resulted in the decrease of exogenous PINK1 (Fig. 2A & 2B). To further investigate whether BAG5 regulated the degradation of exogenous PINK1, we examined the stability of exogenous PINK1 by treatment with cycloheximide to block total protein synthesis in cells stably expressing either EGFP-BAG5 or EGFP alone. HEK-293 cells that were transfected with EGFP-BAG5 vectors showed a decreased degradation of exogenous PINK1 protein after cycloheximide treatment compared with cells transfected for EGFP alone (Fig. 2C & 2D).

### BAG5 stabilizes PINK1 by decreasing the ubiquitination of PINK1

**Figure 3:**
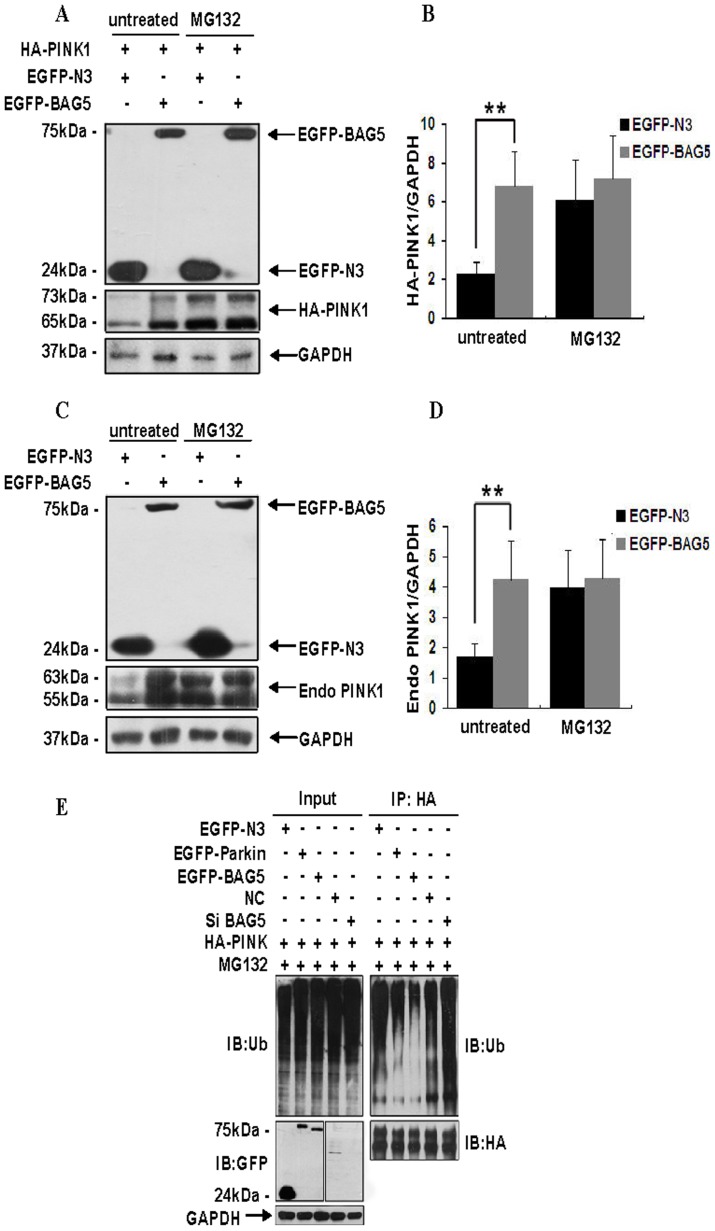
BAG5 inhibited proteasomal degradation of PINK1. **A:** HEK-293 cells overexpressing HA-PINK1 and EGFP or EGFP-BAG5 were treated with the proteasome inhibitor MG132 for 12 h. Cells were lysed, and the extracts were analyzed using specified antibodies against GFP, HA and GAPDH. **B:** Quantitative data from A were shown. The data represented mean ± SE (n = 5; **, P<0.01; Student's t test). **C:** HEK-293 cells overexpressing EGFP or EGFP-BAG5 were treated with the proteasome inhibitor MG132 for 12 h. The cell extracts were analyzed using specified antibodies against GFP, PINK1 and GAPDH. **D:** Quantitative data from C were shown. The data represented mean ± SE (n = 5; **, P<0.01; Student's t test). **E:** HEK-293 cells were co-transfected with HA-PINK1 and EGFP, EGFP-Parkin, EGFP-BAG5, negative control siRNA or BAG5 siRNA. The cells were treated with or without 10 µM MG132 for 12 h and subject to immunoprecipitation using rabbit polyclonal antibodies against HA. Inputs and immunoprecipitants were subject to immunoblot analysis using anti-Ub, anti-EGFP, anti-HA or anti-GAPDH antibody.

Because previous studies had shown that PINK1 was mainly degraded via UPS, we hypothesized that BAG5 may attenuate the proteasomal degradation of PINK1. First, we co-transfected HEK 293 cells with HA-PINK1 and EGFP-BAG5 or HA-PINK1 and EGFP for 24 h, then we treated HEK cells with or without MG132, a proteasomal inhibitor, for about 12 h. The level of HA-PINK1 was markedly increased in cells co-transfected with EGFP-BAG5, but not in cells co-transfected with EGFP tag alone without MG132. However, in the presence of MG132, no differences were observed between the two groups (Fig. 3A & 3B). Similar experiments were performed to determine the effects of BAG5 on the ubiquitin–proteasome degradation of exogenous PINK1. Overexpression of EGFP-BAG5 also inhibited exogenous PINK1 degradation that could be blocked by MG132 (Fig. 3C & 3D). As stability of PINK1 promoted by BAG5 could be inhibited by MG132, the results suggested that BAG5 stabilized PINK1 by interfering with its degradation via UPS. We next examined whether overexpression of BAG5 could decrease the ubiquitination of PINK1. In the presence of MG132, overexpression of EGFP-BAG5 significantly decreased the ubiquitination of PINK1 (Fig. 3E). Similar results were obtained in cells co-transfected with EGFP-Parkin and HA-PINK1 that were served as a positive control. Furthermore, we found that knockdown of BAG5 significantly increased the ubiquitination of PINK1 (Fig. 3E). Thus, our data showed that BAG5 stabilized PINK1 by decreasing ubiquitination of PINK1.

### BAG5 protein rescued MPP^+^-induced mitochondrial dysfunction by up-regulating PINK1

**Figure 4:**
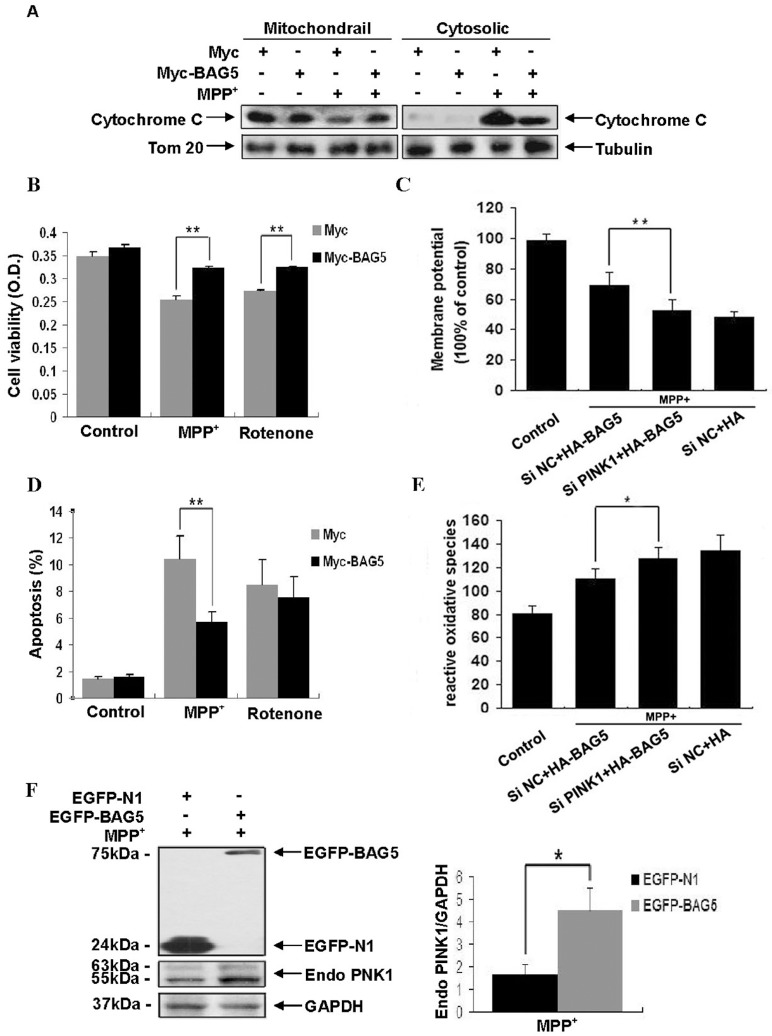
BAG5 protected against MPP^+^- or rotenone-induced mitochondrial damage by up-regulating PINK1. **A:** BAG5 inhibited MPP^+^-induced mitochondrial release of cytochrome c. Stable cell lines overexpressing Myc or Myc-BAG5 were treated with MPP^+^ (250 µM) for 48 h. Immunoblot analysis was performed to detect the subcellular distribution of cytochrome c. **B:** The number of viable cells was determined by MTT assay, which indicated that MPP^+^ or rotenone all induced a significant cell death in control cells, moreover, MPP^+^ and rotenone-induced cell death was significantly inhibited in stable cell lines overexpressing Myc-BAG5. Quantitative data represented mean ± SE (n = 4; **, P<0.01; Student's t test). **C:** Mitochondrial membrane potential was estimated in transfected cells with treatment of MPP^+^. After knocking down endogenous PINK1, Mitochondrial membrane potentials of HA-BAG5 stable cell line was significantly increased compared with si NC transfected stable cell line. Quantitative data represented mean ± SE (n = 4; *, P<0.05; Student's t test). **D:** Cellular apoptosis was estimated in stable cell lines with or without treatment of MPP^+^ or rotenone. Under MPP^+^ treatment, apoptosis of the stable cell line overexpressing Myc-BAG5 was significantly decreased compared with Myc stable cell line. Quantitative data represented mean ± SE (n = 4; **, P<0.01; Student's t test). **E:** ROS production was detected in stable cell lines with treatment of MPP^+^. After knocking down endogenous PINK1, ROS production in the stable cell line overexpressing HA-BAG5 was significantly increased compared with si NC transfected stable cell line. Quantitative data represented mean ± SE (n = 4; *, P<0.05; Student's t test). **F:** Overexpression of EGFP-BAG5 could up-regulate exogenous PINK1 in the MPP^+^ treated cells. **G:** quantitative analysis of protein levels in F, results were represented as the mean ± S.E (n = 4; *, p<0.05; Student's t test).

To investigate the protective effects of BAG5 on mitochondrial function, stable HEK-293 cell lines expressing Myc-BAG5 or Myc were treated with or without MPP^+^ (250 µM) for 48 h. Then we detected the changes of mitochondrial release of cytochrome c, cell death, and apoptosis to evaluate mitochondria dysfunction. And we further investigated if knocking down of PINK1 could abolish the protective effect of BAG5. Stable SH-SY5Y cell lines expressing HA-BAG5 or HA were transiently transfected with PINK1 siRNA or negative control siRNA for 48 h, then treated with MPP^+^ (250 µM) for 48 h. In our studies, overexpression of BAG5 in HEK 293 cells inhibited MPP^+^-induced mitochondrial release of cytochrome c into the cytosol (Fig. 4A). Treatment of HEK 293 cells with MPP^+^ (250 µM) caused the increase of cell death and apoptosis. However, overexpressing of BAG5 significantly reduced MPP^+^ -induced cell death (Fig. 4B) and apoptosis (Fig. 4D). The results suggested BAG5 exerted neuroprotective effects by protecting mitochondria against MPP^+^-induced oxidative stress damage. Further investigates showed knocking down of PINK1 in stable SH-SY5Y cell lines significantly inhibited protective effect of BAG5 on MPP^+^-induced mitochondrial damage. Decrease of PINK1 levels in the HA-BAG5 overexpressing cell line caused the collapse of mitochondrial membrane potential (Fig. 4C) and ROS generation (Fig. 4E). Cells expressing EGFP-BAG5 displayed increasing in the level of exogenous PINK1 compared with cells expressing only EGFP under MPP^+^ treatment (Fig. 4F & 4G), which suggested that BAG5 might protect against MPP^+^-induced mitochondrial damage by up-regulating PINK1.

### Decrease of PINK1 levels induced by suppression of PINK1 expression or exposing to neurotoxin MPP^+^ results in a strong upregulation of BAG5 and increased mitochondrial localization of BAG5

**Figure 5:**
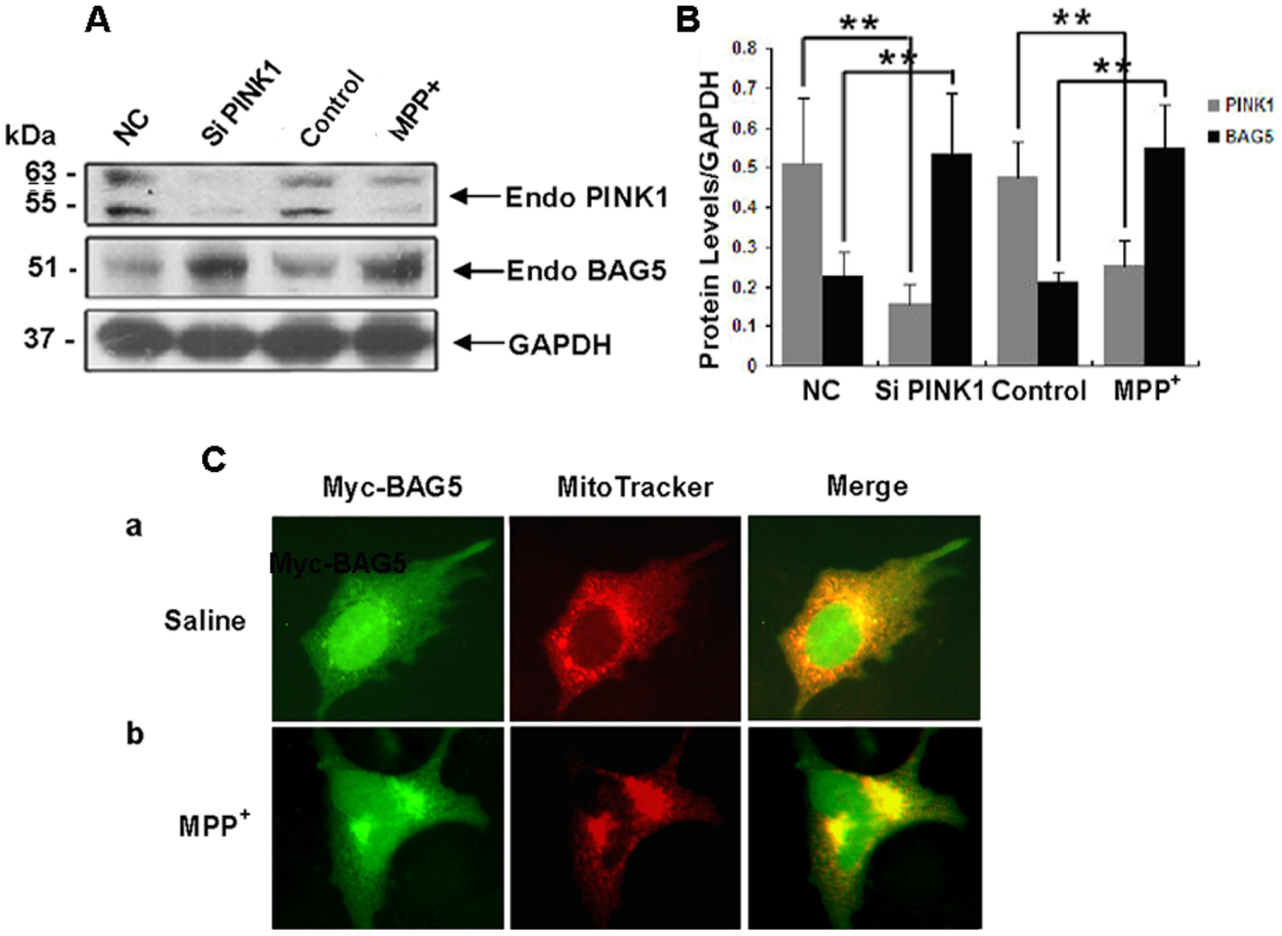
Silencing of PINK1 expression or exposing to MPP^+^ led to increase in the level of exogenous BAG5 in PC12 cells. **A:** PC12 cells were divided into four groups, the first two groups were transfected with negative control siRNA (NC) or PINK1 siRNA for 48 h, and the last two groups were treated with or without MPP^+^ for 48 h. Cells extracts were collected after transfection and subjected to immunoblot analysis. Suppression of PINK1 expression by PINK1-targeting siRNAs significantly enhanced exogenous BAG5 levels, and decrease of exogenous PINK1 induced by neurotoxin MPP^+^ also resulted in a significant increase of exogenous BAG5. **B:** Quantitative data from A were shown. The data represented mean ± SE (n = 4; **, P<0.01; Student's t test). **C:** MPP^+^ led to increased BAG5 mitochondrial localization. HEK-293 cells were transfected with Myc-BAG5 and RFP-Mito, 24 h after transfection, the cells were treated with or without MPP^+^ (250 µM) for another 48 h. After fixation, Myc tagged proteins staining were revealed by anti-Myc monoclonal antibody (green) and compared with MitoTracker (red). (a) Merged green and red images showed partial co-localization of BAG5 and mitochondria markers under control conditions; (b) increased mitochondrial co-localization of BAG5 and MitoTracker when cells were treated with MPP^+^ for 48 h.

Based on our current finding, we wondered whether decrease of PINK1 resulted in feedback upregulation of BAG5. We first transiently transfected PC12 cells with PINK1 siRNA or negative control siRNA for 48 h. In PINK1 knockdown cells, the levels of exogenous BAG5 were significantly increased (Fig. 5A & 5B). Next, PC12 cells were incubated with or without MPP^+^ (250 µM) for 48 h, then cells extracts were collected and subjected to immunoblot analysis. Under MPP^+^ treatment, the level of exogenous PINK1 was significantly decreased, whereas exogenous BAG5 was markedly increased (Fig. 5A & 5B). These results suggested that decrease of PINK1 resulted in feedback upregulation of BAG5, which increased the stability of PINK1. We co-transfected HEK 293 cells with Myc-BAG5 and RFP-Mito, the specific organelle markers which labeled mitochondria. The results confirmed that BAG5 partially distributed in the mitochondria (Fig. 5C). Under MPP^+^ treatment, mitochondrial distribution of BAG5 was significantly increased, suggesting that BAG5 was recruited onto the mitochondria to stabilize PINK1 following the induction of MPP^+^ (Fig. 5C).

### Genetic ablation of mouse PINK1 results in increase of BAG5 in SNc

**Figure 6:**
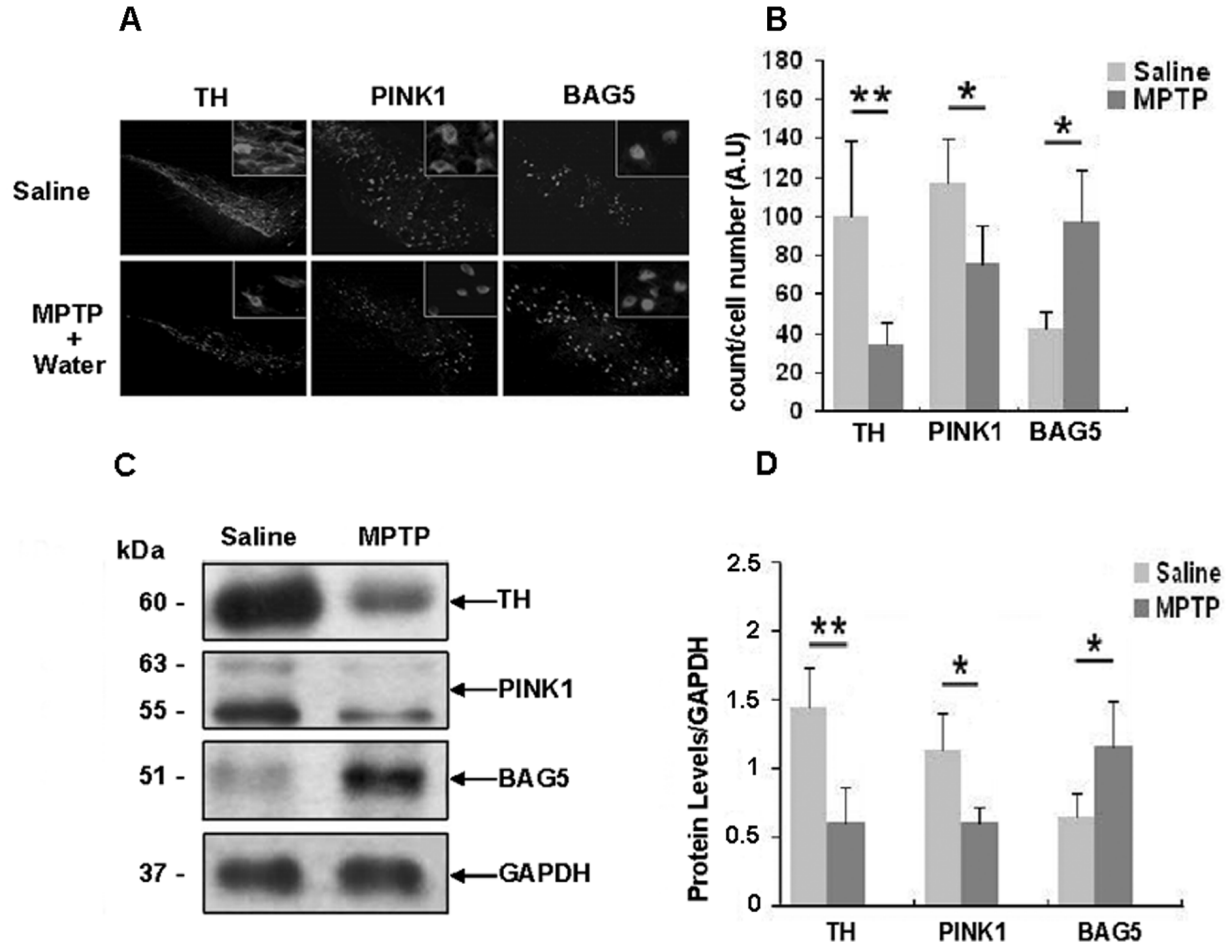
Increased levels of BAG5 protein in SNpc of PINK1−/− Mice. **A:** Immunohistochemistry showed images of TH, PINK1 and BAG5- immunopositive neurons in the SNpc of control and PINK1−/− mice. **B:** Quantitative analysis of TH, PINK1 and BAG5 positive neurons from the SNpc of wild-type mice and PINK1−/− mice was shown as the mean ± S.E (n = 6; *, p<0.05; **, p<0.01; Student's t test). **C:** Immunoblot analysis showed endogenous TH, PINK1 and BAG5 protein expression in the SNpc of control and PINK1−/− mice. **D:** Quantitative analysis of protein levels in C, representative results were shown as the mean ± S.E (n = 6, *, p<0.05, **, p<0.01, Student's t test).

To validate our observations that suppression of PINK1 expression significantly enhanced exogenous BAG5 in vitro, we further investigated whether it could happen in vivo. Accordingly, we generated the PINK1-null mouse to detect the changes of BAG5 in dopaminergic neurons in SNc. The midbrain was sectioned, and the number of tyrosine hydroxylase (TH), PINK1 and BAG5-immunopositive neurons in SNc was assessed. We found that the expression of BAG5 was increased in SNc of PINK1-null mouse (Fig. 6).

### MPTP treatment results in increase of BAG5 in SNc

**Figure 7:**
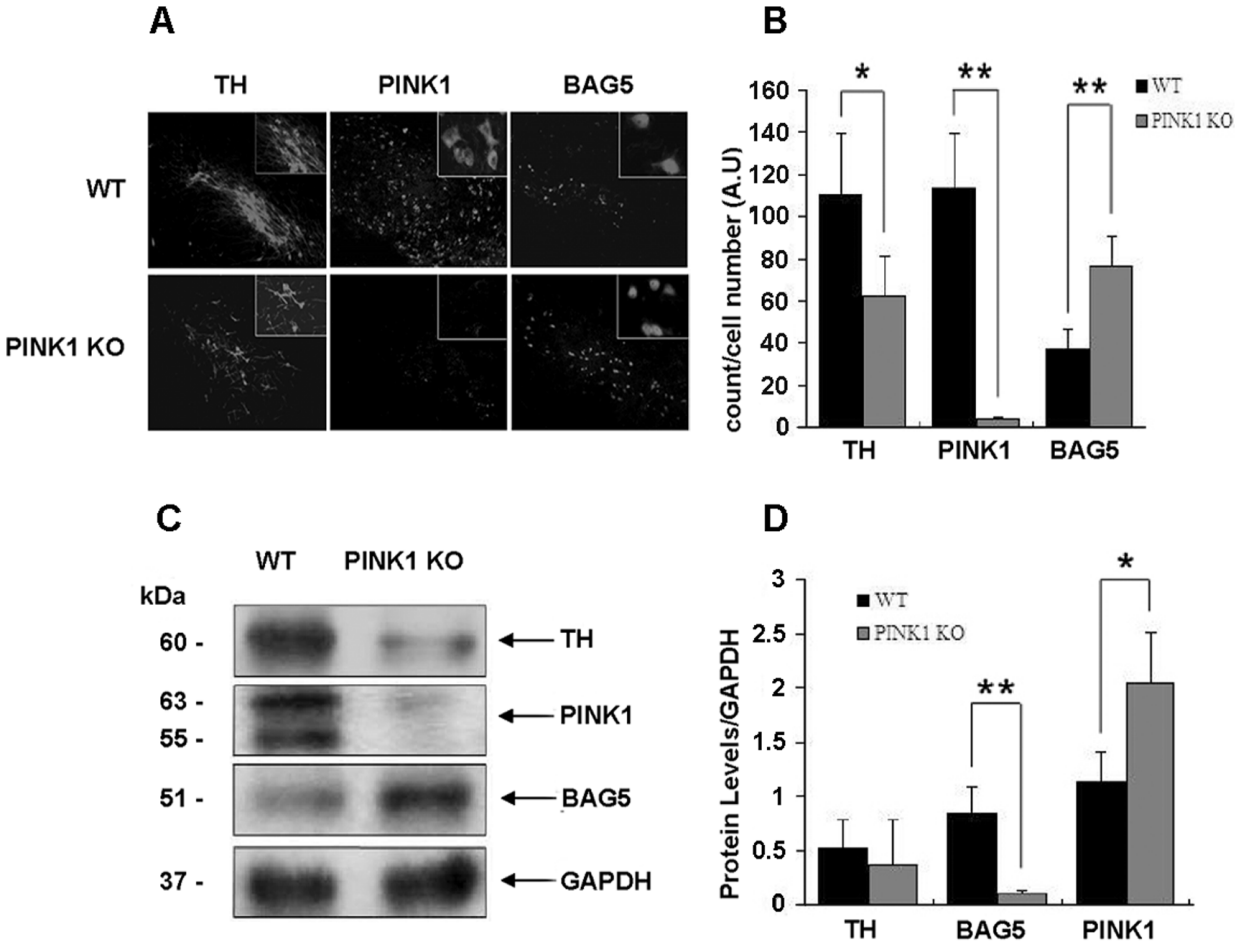
Expression of BAG5 was increased in the SNpc of MPTP model of PD. **A**: Representative photomicrographs of TH, PINK1, BAG5-labeled sections of the SNpc. MPTP reduced the content of TH and PINK1 inside the dopaminergic neurons of the SNpc, but increased the BAG5 immunoreactivity. **B**: quantitative analysis of A were shown as the mean ± S.E. (n = 8; *, p<0.05; **; p<0.01; Student's t test). **C**: Western blot showed endogenous TH, PINK1 and BAG5 protein expression in the SNpc. **D**: quantitative analysis of protein levels in C were shown as the mean ± S.E. (n = 8; *, p<0.05; **, p<0.01; Student's t test).

In light of the fact that MPP^+^ treatment led to an increase in the levels of exogenous BAG5 in PC12 cells, we checked whether BAG5 expression was up-regulated in the SNpc of MPTP model of PD. C57BL mice were administrated with MPTP (25 mg/kg, i.p., per day) for 8 days, followed by a further 3 days resting period to allow for the full conversion of MPTP to its active metabolite, MPP^+^ [22]. At day 12, either rasagiline mesylate (0.08 mg/kg, p.o., per day) or water were administered for 10 days, reaching a total treatment period of 22 days. Another group consisted of mice receiving saline for 8 days and water for 12 days as control. We conducted immunohistochemical analysis and western blot analysis to evaluate the changes of the protein levels. The results showed that MPTP caused a significant reduction of TH and PINK1-positive cells, whereas BAG5-positive cells were significantly increased (Fig. 7).

## Discussion

Mutations in *PINK1* gene cause PARK6 autosomal recessive early-onset parkinsonism, and mutations of *PINK1* have also been noted in idiopathic PD patients, suggesting mutations of *PINK1* are associated with PD. Previous studies in cell lines and animal models demonstrated that PINK1 acted as a cytoprotective protein and that aberrant regulation and inactivation of PINK1 induced apoptosis in dopaminergic cells [23]–[27], however, the exact mechanism about the neuroprotective effects of PINK1 is not yet clear. More and more experimental evidence strongly suggests that interactions between PINK1 and its upstream or downstream proteins may be important for the neuroprotective function of PINK1. To date, no protein is identified to involve in degradation of PINK1 except Parkin. Previous study demonstrated that Parkin stabilizes PINK1 by interfering with its degradation via the UPP and operated through a common pathway with PINK1 in the pathogenesis of early-onset PD [9]–[14].

Here, we present BAG5, a member of the BAG family proteins, as new binding partner for PINK1, with important consequences for PINK1 stability. New roles for the BAG proteins continue to be discovered, such as mediating proper protein folding and involving in proteasome-mediated protein degradation and autophagy [28]. BAG1 reportedly acts as a shuttle protein to deliver misfolded substrates to the proteasome for degradation [29], [30]. BAG-2 has been demonstrated to inhibit the ubiquitin ligase activity of CHIP by abrogating the CHIP/E2 cooperation [31]. Little is known about the functions of BAG5 other than its function as the nucleotide exchange factor of Hsp70, however, a recent study has shown that BAG5 interacts directly with Parkin and inhibit Parkin E3 ligase activity, which inhibits parkin-mediated ubiquitinylation of synphilin [20]. This study suggests that BAG5 also involves in proteosome-mediated protein degradation, which is associated with Parkinson's disease.

In our study, we have demonstrated a physical interaction between PINK1 and BAG5. This interaction depended on the kinase domain of PINK1 and the BD of BAG5. In cells overexpressing BAG5, PINK1 levels increased significantly, and degradation of the PINK1 was attenuated. In BAG5 knockdown cells, PINK1 levels decreased and the ubiquitination of PINK1 increased. Previous reports showed PINK1 degradation by the proteasome [15], [32], [33]. To our knowledge, Parkin is one of the few proteins which could regulate the degradation of PINK1. Interestingly, in contrast to its E3-ligase activity, Parkin stabilizes PINK1 by interfering with its degradation through the UPS [11]. Our data revealed that BAG5 inhibited PINK1 degradation through direct interaction with PINK1 via the UPP, it is therefore possible that BAG5 stabilizes PINK1 protein by inhibiting the E3-ligase of PINK1 or unknown PINK1-ubiquitination factors.

Our findings suggested that physiological state of BAG5 played an important role in stabilizing PINK1 by its decreasing the ubiquitination of PINK1. We further used cell culture models and animal models to investigate the interaction between BAG5 and PINK1 under pathologic status. PINK1 knockdown cells and MPP^+^ cell models were produced to simulate the decrease of PINK1 levels in pathological conditions. An interesting outcome of the cellular experiments was that decrease of PINK1 levels induced by suppression of PINK1 or exposing to MPP^+^ resulted in a strong upregulation of BAG5 levels and increased mitochondrial localization of BAG5. Moreover, overexpression of BAG5 could restore the exogenous PINK1 levels under MPP^+^ treatment. Thus, we supposed that the decrease of PINK1 levels in pathological conditions might result in feedback upregulation of BAG5 levels in vitro. Regulating mitochondrial structure and function was one of the important downstream functions of PINK1. Our study found that MPP^+^ and rotenone-induced mitochondria dysfunction characterized by mitochondrial membrane potential loss, cytochrome c release and elevation of ROS level could be rescued by BAG5 protein. Taken these data together, we presumed that BAG5 might protect mitochondrial function against MPP^+^ and rotenone through up-regulating endogenous PINK1 protein.

In vivo, we used the PINK1-null mouse and MPTP mouse model to further validate our findings in vitro. BAG5 expression was significantly increased in SNpc of PINK1-null mouse and MPTP mouse, with significant reduction of TH and PINK1-positive cells. The results also indicated that the decrease of PINK1 levels in pathological conditions might result in feedback upregulation of BAG5 levels in vivo.

In conclusion, we reported a novel role for BAG5 as a modulator of PINK1 protein degradation. We had shown a direct and functional interaction between PINK1 and BAG5, which was vitally important for maintaining mitochondrial physiological function and protecting mitochondria against oxidative stress. The characterization of the interaction between PINK1 and BAG5 under physiological status or pathological state will lead to an improved understanding of the pathogenesis of Parkinson's disease and, ultimately, possibly to novel therapeutic approaches.

## Materials and Methods

### Plasmid constructs

The mammalian expression plasmid pKH3-HA-PINK1 was a kind gift from Dr Bin Li (University of Science & Technology of China), pGEX-5X-1-PINK1 from Dr Qingsong Hu (University of Science & Technology of China), EGFP-Parkin and V1664 pLPS-3′RFP-mito from Dr Dong Chen (University of Science & Technology of China). Full-length BAG5 cDNA was amplified from a human fetal brain library (Invitrogen) using the primers 5′-cggaattctatgcgtttccattggttaccc-3′ and 5′-cgcggatccgtactcccattcatcaga-3′ and inserted into pcDNA3.1(+)/myc-HisA vector at BamHI/EcoRI sites. For the construct of pBK-CMV-BAG5, full length BAG5 cDNA was excised from pcDNA3.1(+)/myc-HisA-BAG5 with BamHI/EcoRI sites and subcloned into pBK-CMV vector. pEGFP-BAG5 was constructed by subcloning a fragment excised from pcDNA3.1(+)/myc-HisA-BAG5 into pEGFP-N3 vector at BamHI/EcoRI sites. pGEX-5X-1-BAG5 was constructed by subcloning a fragment excised from pBK-CMV-BAG5 into pGEX-5X-1 vector at EcoRI/XhoI sites. The deletions of pGEX-5X-1-BAG5 were generated by with following primers: 5′-cggaattcttgagcgagccgttt-3′ and 5′-gcgtcgactcaaatcctctccagtttc-3′ for pGEX-5X-1-BAG5(9–86); 5′-cggaattctaacaaaacagctttttg-3′ and 5′-gcgtcgactcatccagttttaacatgtg-3′ for pGEX-5X-1-BAG5(87–181); 5′-cggaattcggaaaaatctccttg-3′ and 5′-gcgtcgactcacacacaggataagtg-3′ for pGEX-5X-1-BAG5(182–260); 5′-cggaattcggccggacagaaatcag-3′ and 5′-gcgtcgactcattctgttttggagctcag-3′ for pGEX-5X-1-BAG5(275–350); 5′-cggaattcaaaaacccctgcatc-3′ and 5′-gcgtcgactcacagctcttccagcc-3′ for pGEX-5X-1-BAG5(365–442); then the deletions were inserted into pGEX-5X-1 vectors at EcoRI/SalI sites. The deletions of pGEX-5X-1-PINK1 were generated by with following primers: 5′-cggaattcatggcggtgcgacaggcg-3′ and 5′-ccctcgagaggccccggcttgctttt-3′ for pGEX-5X-1-PINK1(1–140); 5′- cggaattcatgtatctgatagggcag-3′ and 5′-ccctcgagtagatgaagcacatttgc-3′ for pGEX-5X-1-PINK1(156–509); then the deletions were inserted into pGEX-5X-1 vectors at EcoRI/SalI sites. All constructs were sequenced to confirm their fidelity.

### In vitro binding assay and Immunoprecipitation

To test for interactions between BAG5 domains and PINK1 domains in vitro, binding assays were performed. An aliquot of protein from the soluble fraction of E. coli crude extract were incubated with 30 µl of glutathione agarose beads (Pharmacia) for 30 min on ice. After washing three times with 1×PBS, beads bound with GST-BAG5 or GST-PINK1 were incubated with 50 µg of protein from the supernatants of E. coli crude extract containing recombinant His-PINK1 or His-BAG5 expressed by pET-21a-PINK1 or pET-21a-BAG5 in 0.25 ml HNTG-buffer [20 mM HEPES-KOH (pH 7.5), 100 mM NaCl, 0.1% Triton X-100 and 10% glycerol] for 2 h at 4°C. Samples were washed twice with 1×PBS, followed by addition of His-PINK1 or His-BAG5 for 2 h on ice. Bound proteins were eluted from the beads by boiling in SDS sample buffer and detected by immunoblot analysis. Crude cell lysates were sonicated in TSPI buffer [50 mM Tris–HCl (pH 7.5), 150 mM NaCl, 1 mM EDTA, 1 mg/ml aprotinin, 10 mg/ml of leupeptin, 0.5 mM Pefabloc SC, 10 mg/ml of pepstatin, 1% NP-40]. Cellular debris was removed by centrifugation at 12 000 g for 20 min at 4°C. The supernatants were incubated with the anti-bodies in 0.01% BSA for 4 h at 4°C. After incubation, protein G Sepharose (Roche) was used for precipitation. The beads were washed with TSPI buffer seven times, and proteins were eluted with SDS sample buffer for immunoblot analysis.

### Cell culture, transfections, drug treatments and RNA interference

HEK-293, PC12, SH-SY5Y cells (purchased from the Type Culture Collection of the Chinese Academy of Sciences, Shanghai, China) were cultured in Dulbecco's modified Eagle's medium (DMEM) (GIBCO) containing 10% newborn calf serum (NCS) (GIBCO). Transfections were performed using Lipofectamine 2000 (Invitrogen) according to the manufacturer's instructions. MG132 was obtained from Calbiochem and Cycloheximide (CHX) was purchased from Sigma. A stable HEK-293 cell line expressing Myc-BAG5 was cultured in DMEM with 10% NCS and 200 mg/ml G418 (GIBCO). According to the manufacturer's instructions (Invitrogen), 50 pmol of each siRNA was transfected using Oligofectamine, Oligo RNA were purchased from GenePharma (Shanghai, China) and had the following sequences: Si PINK1 sense, 5′-GCCAUCUUGAACACAAUGATT-3′, Si PINK1 antisense, 5′-UCAUUGUGUUCAAGAUGGCTT-3′; Si BAG5 sense, 5′-GGAGAUAUUCAGCAAGCUATT-3′, Si BAG5 antisense, 5′-UAGCUUGCUGAAUAUCUCCTT-3′; Si control sense, 5′-UUCUCCGAACGUGUCACGUdTdT-3′, Si control antisense, 5′-ACGUGACACGUUCGGAGAAdTdT-3′.

### Immunohistofluorescence, Immunocytochemistry and antibodies

HEK-293 cells were washed with PBS and fixed with 4% paraformaldehyde for 5 min at room temperature. Then cells were treated with 0.25% Triton X-100 for 15 min and blocked by 4% FBS for 20 min, then incubated overnight at 4°C with the primary antibody following by an incubation with FITC donkey anti-mouse secondary antibodies (1∶200 dilution; Santa Cruz Biotechnology). All images were collected using fluorescent microscopy. Free-floating sections were washed 3 times with 0.01 M PBS, then tissue sections were previously dehydrated, treated with 0.5% (v/v) H_2_O_2_ in methanol for 30 min, unspecific binding sites were blocked by 0.01 M PBS, 5% bovine serum albumin (BSA) and 0.3% Triton X-100 at room temperature for 2 h. Immunohistochemical staining experiments were performed using indicated antibodies and detected with Rho-conjugated donkey anti-mouse or rabbit secondary antibodies (1∶5000 dilution; Santa Cruz Biotechnology). The following primary antibodies were used: mouse monoclonal antibodies against BAG5 (1∶700 dilution; Abcam), Tubulin (1∶700 dilution; Santa Cruz), HA (1∶1000 dilution; Santa Cruz), GFP (1∶1000 dilution; Santa Cruz), GAPDH (1∶2000 dilution; Chemicon), Ub (1∶300 dilution; Santa Cruz), Myc (1∶1000 dilution; Santa Cruz), cytochrome (1∶300 dilution; Santa Cruz), Tom20 (1∶500 dilution; Sigma); rabbit polyclonal antibodies against PINK1 (1∶500 dilution; Abcam), HA (1∶500 dilution; Abcam), TH (1∶700 dilution; Abcam). The secondary antibodies, sheep anti-mouse IgG-HRP antibody (1∶200 dilution; Santa Cruz) and anti-rabbit IgG-HRP antibody (1∶200 dilution; Santa Cruz) were used. The proteins were visualized using an ECL detection kit (Amersham Pharmacia Biotech).

### Cell Viability

Cell viability was measured by using the 3-(4,5-Dimethylthiazol-2-yl)-2,5-diphenyltetrazolium bromide (MTT) assay, which was based on the conversion of MTT to formazan by mitochondrial and cytosol dehydrogenases [34], [35].

### Measurement of mitochondrial membrane potential

Mitochondrial membrane potential was measured by the incorporation of a cationic fluorescent dye rhodamine 123 as previously described [36]. After 30 min incubation in DMEM with 10% FBS, the cells were changed to serum-free medium containing 10 µmol rhodamine 123 and incubated at 37°C for 15 min. The cells were then collected and the fluorescence intensity was analyzed with a Hitachi F-4500 spectrophotofluorimeter (490 nmexcitation and 515 nmemission).

### Measurement of intracellular reactive oxidative species

Cell-permeable nonfluorescent 2′7′-dicholorodihydrofluorescein diacetate (DCFH-DA) (Invitrogen) was loaded directly into the growth plate to equal a final concentration of 10 µM solution in DMEM. Samples were then analyzed immediately on a FACS Calibur flow cytometer utilizing excitation at 488 nm and fluorescein isothiocyanate (FITC) filter detection parameters. Cell Quest Pro software (BD Biosciences) was used for analysis to produce histogram plots and median peak values.

### Animals and treatments

Ten-week-old, male, C57BL mice were given free access to food and tap water and were caged individually under controlled temperature (23±2°C) and humidity (55±5%) with an artificial light cycle. The mice were divided into three groups of 10 mice (saline/water, MPTP/water and MPTP/rasagiline),and were subjected to a subacute MPTP regimen. Mice in Group 1 received intraperitoneal injection of 25 mg/kg saline daily for 7 days, followed by a further 3 days resting period, and then water were administered for 10 days. Mice in Group 2 received intraperitoneal injection of 25 mg/kg MPTP daily for 7 days, followed by a further 3 days resting period, and then water were administered for 10 days. Mice in Group 3 received intraperitoneal injection of 25 mg/kg MPTP daily for 7 days, followed by a further 3 days resting period, and then rasagiline were administered for 10 days. Mice were sacrificed under anesthesia by i.p. injection of sodium pentobarbital (50 mg/kg). Assessment of SNpc was performed 23 days after the start of the MPTP dosing regimen. The PINK1–deficient mouse line has been described previously, heterozygous of PINK1–deficient mouse on a 129×C57BL/6 mixed background were bred to generate Pink1-null mice and their WT littermate controls for experiments [32]. All procedures related to animal care and treatment were conducted with the approval of the Animal Welfare Committee of University of Science and Technology of China; and according to the guidelines National Research Council Institute for Laboratory Animal Research Guide for the Care and Use of Laboratory Animals.
